# Reply to comments and questions of Dr. Correale et al. about our review concerning CTEPH

**DOI:** 10.1007/s12471-015-0668-7

**Published:** 2015-02-21

**Authors:** Bastiaan E. Schölzel, Repke J. Snijder, Johannes J. Mager, Hendrik W. van Es, Herbert W.M. Plokker, Herre J. Reesink, Wim J. Morshuis, Martijn C. Post

**Affiliations:** 1 Department of Cardiology, Amphia Hospital, Molengracht 21, 4818 CK Breda, The Netherlands; 2 Department of Cardiology, St. Antonius Hospital, Koekoekslaan 1, 3435 CM Nieuwegein, The Netherlands; 3 Department of Pulmonology, St. Antonius Hospital, Koekoekslaan 1, 3425 CM Nieuwegein, The Netherlands; 4 Department of Radiology, St. Antonius Hospital, Koekoekslaan 1, 3425 CM Nieuwegein, The Netherlands; 5 Department of Cardio-Thoracic Surgery, St. Antonius Hospital, Koekoekslaan 1, 3425 CM Nieuwegein, The Netherlands

We would like to thank Dr. Correale et al. for their interest and valuable comments on our review regarding the diagnostic approach to chronic thromboembolic pulmonary hypertension (CTEPH) and the available surgical and medical therapeutic options [1]. They ask for further comments on congenital abnormalities causing hypercoagulability in these patients.

The prevalence of CTEPH after acute pulmonary embolism is estimated at 0.1–4.0 % after 2 years [2−5]. It is still not known why some patients develop CTEPH after acute pulmonary embolism and others do not. The risk of developing CTEPH is increased in patients who have recurrent venous thromboembolism, large perfusion defects and echocardiographic signs of pulmonary hypertension at the initial presentation [6]. Interestingly, the development of CTEPH is not associated with common risk factors for venous thromboembolism, such as factor V Leiden, factor II mutation, deficiency of antithrombin, protein C and protein S or a prothrombin G20210A gene mutation [7]. The prevalence of these factors is low in CTEPH and some of them seem to occur with similar frequency among patients with CTEPH and the general population. There are two exceptions. The presence of antiphospholipid antibodies and Lupus anticoagulant is found in 10–20 % of the CTEPH patients and antiphospholipid antibodies predispose to acute venous thromboembolism and in some cases even recurrent pulmonary embolism [6,8−10].The study of D’Armini et al. compared 28 patients with high levels of antiphospholipid antibodies with 156 patients with low level or absence of antiphospholipid antibodies who all underwent pulmonary endarterectomy. There was no difference between the two groups after surgery in terms of mortality and major complications. However, the patients with high levels of antiphospholipid antibodies had significantly more transient neurological complications postoperatively [11].

Several studies demonstrated increased levels of FVIII in CTEPH patients [12−14]. One study investigated the effect of pulmonary endarterectomy on FVIII levels, and found no change after surgery. Interestingly, the level of FVIII decreases after medical treatment of pulmonary arterial hypertension (PAH) [15]. However, the exact mechanism of how these factors contribute to CTEPH remains unknown. To our knowledge, there are no studies comparing methylenetetrahydrofolate reductase (MTHFR) C677T polymorphism between CTEPH patients and healthy controls. Furthermore, MTHFR polymorphism is not associated with PAH [16].

Routine screening for thrombophilia seems only reasonable when it has an impact on the prognosis, treatment or outcome of CTEPH. We must keep in mind that all patients receive life-long anticoagulant treatment. In the literature, only high levels of antiphospholipid antibodies were demonstrated to influence postoperative outcome after pulmonary endarterectomy. Therefore, according to the guideline, we think that screening for the antiphospholipid syndrome is reasonable [17].

## References

[1] Schölzel BE, Snijder RJ, Mager JJ (2014). Chronic thromboembolic pulmonary hypertension. Neth Heart J.

[2] Pengo V, Lensing AW, Prins MH (2004). Thromboembolic Pulmonary Hypertension Study Group. Incidence of chronic thromboembolic pulmonary hypertension after pulmonary embolism. N Engl J Med.

[3] Ribeiro A, Lindmarker P, Johnsson H, Juhlin-Dannfelt A, Jorfeldt L. (1999). Pulmonary embolism: one-year follow-up with echocardiography Doppler and five-year survival analysis. Circulation.

[4] Moser KM, Auger WR, Fedullo PF (1990). Chronic major-vessel thromboembolic pulmonary hypertension. Circulation.

[5] Becattini C, Agnelli G, Pesavento R (2006). Incidence of chronic thromboembolic pulmonary hypertension after a first episode of pulmonary embolism. Chest.

[6] Lang IM, Pesavento R, Bonderman D, Yuan JX (2013). Risk factors and basic mechanisms of chronic thromboembolic pulmonary hypertension: a current understanding. Eur Respir J.

[7] Konstantinides SV, Torbicki A, Giancarlo Agnelli G, The Task Force for the Diagnosis and Management of Acute Pulmonary Embolism of the European Society of Cardiology (ESC)Endorsed by the European Respiratory Society, (ERS) (2014). 2014 ESC Guidelines on the diagnosis and management of acute pulmonary embolism. Eur Heart J.

[8] Wolf M, Boyer-Neumann C, Parent F (2000). Thrombotic risk factors in pulmonary hypertension. Eur Respir J.

[9] Morris TA (2013). Why acute pulmonary embolism becomes chronic thromboembolic pulmonary hypertension: clinical and genetic insights. Curr Opin Pulm Med.

[10] Bonderman D, Wilkens H, Wakounig S (2009). Risk factors for chronic thromboembolic pulmonary hypertension. Eur Respir J.

[11] D’Armini AM, Totaro P, Nicolardi S, et al. Impact of high titre of antiphospholipid antibodies on postoperative outcome following pulmonary endarterectomy. Interact Cardiovasc Thorac Surg. 2010;10:418–22. 10.1510/icvts.2009.221630 19934162

[12] Bonderman D, Turecek PL, Jakowitsch J (2003). High prevalence of elevated clotting factor VIII in chronic thromboembolic pulmonary hypertension. Thromb Haemost.

[13] Bonderman D, Jakowitsch J, Adlbrecht C (2005). Medical conditions increasing the risk of chronic thromboembolic pulmonary hypertension. Thromb Haemost.

[14] Wong CL, Szydlo R, Gibbs S, Laffan M. (2010). Hereditary and acquired thrombotic risk factors for chronic thromboembolic pulmonary hypertension. Blood Coagul Fibrinolysis.

[15] Friedman R, Mears JG, Barst RJ (1997). Continuous infusion of prostacyclin normalizes plasma markers of endothelial cell injury and platelet aggregation in primary pulmonary hypertension. Circulation.

[16] Day RW, Mack GK, Barker AM, Rees TQ, Jorgensen LO, Botto LD. Prevalence of Variants in Methylenetetrahydrofolate Reductase and the Severity of Pulmonary Vascular Disease. Pediatr Cardiol. 2014 Oct 11. [Epub ahead of print]. 10.1007/s00246-014-1044-x 25304246

[17] Task Force for Diagnosis and Treatment of Pulmonary Hypertension of European Society of Cardiology (ESC); European Respiratory Society (ERS); International Society of Heart and Lung Transplantation (ISHLT), Galiè N, Hoeper MM, Humbert M, et al. Guidelines for the diagnosis and treatment of pulmonary hypertension. Eur Respir J. 2009;34:1219–63. 10.1183/09031936.00139009 19749199

